# Expression, Purification and Characterization of Functional Teduglutide Using GST Fusion System in Prokaryotic Cells

**DOI:** 10.34172/apb.2023.064

**Published:** 2022-12-06

**Authors:** Ali Akbar Alizadeh, Saba Rasouli, Omid Jamshidi Kandjani, Salar Hemmati, Siavoush Dastmalchi

**Affiliations:** ^1^Biotechnology Research Center, Tabriz University of Medical Sciences, Tabriz, Iran.; ^2^Pharmaceutical Analysis Research Center, Tabriz University of Medical Sciences, Tabriz, Iran.; ^3^School of Pharmacy, Tabriz University of Medical Sciences, Tabriz, Iran.; ^4^Drug Applied Research Center, Tabriz University of Medical Sciences, Tabriz, Iran.; ^5^Faculty of Pharmacy, Near East University, Po.Box: 99138, Nicosia, North Cyprus, Mersin 10, Turkey.

**Keywords:** Recombinant technology, Peptide, Teduglutide, Affinity chromatography, SBS, Size exclusion chromatography

## Abstract

**Purpose:**

Teduglutide is the first and only FDA-approved drug for long-term treatment of short bowel syndrome (SBS). The current study aimed to present an approach for production of teduglutide using recombinant DNA technology.

**Methods:**

The coding gene for teduglutide was cloned into pGEX-2T vector, where coding sequence for factor Xa cleavage site was added between GST and teduglutide coding genes. The GST-teduglutide protein was overexpressed in *E. coli* BL21 (DE3) strain and affinity purified using glutathione sepharose affinity column.

**Results:**

On-column proteolytic activity of factor Xa followed by size exclusion chromatography resulted in the pure teduglutide. Circular dichroism (CD) spectropolarimetry showed that the produced teduglutide folds into mainly α-helical structure (>50%), as expected. In mass spectroscopy analysis, the fragments of teduglutide resulted by cyanogen bromide cleavage as well as those expected theoretically due to mass fragmentation were identified. The functionality of the produced peptide was evaluated by measuring its proliferative effect on Caco2 intestinal epithelial cells, and the results indicated that produced teduglutide induces cell proliferation by 19±0.30 and 33±7.82 % at 1.21 and 3.64 µM concentrations, respectively, compared to untreated cells.

**Conclusion:**

Teduglutide was successfully expressed and purified and its functionality and structural integrity were confirmed by *in vitro* experiments. We believe that the experimental-scale method presented in the current study can be useful for pilot-scale and also industrial-scale production of teduglutide.

## Introduction

 Short bowel syndrome (SBS) is a debilitating illness in which absorption of nutrients from food is disrupted due to the loss of significant portion of intestine. The clinical manifestations of SBS are diarrhea, steatorrhea, abdominal pain, electrolyte imbalances, dehydration, and malnutrition.^1-3^ The main reasons for SBS are surgical removal of intestine due to Crohn’s disease, cancer, traumatic injuries and dysfunction in blood supply to the intestine or congenital missing or damaged small intestine.^1-3^ This disability was compensated to some extent by secretion of endogenous trophic hormones and peptides such as glucagon-like peptide-2 (GLP-2), which regulate the growth, proliferation^4-6^ and maintenance of cells lining the gastrointestinal tract. In most cases, metabolic and pharmacological interventions are inadequate to cope with malnutrition in SBS patients, and hence, parenteral support is considered for lifesaving. Although effective, parenteral nutrition is faced with the inconvenience and complications such as infections, thrombosis, liver failure and hypo-or hyperglycemia which worsen the quality of patients’ life. Therapeutic choices for SBS patients are growth factors and seven other trophic hormones^7^ and among them; FDA has approved short-term application of human growth hormone, somatropin and L-glutamine in SBS patients with limited efficacy.^5,8^ GLP2 is an endogenous throphic peptide which has indispensable activity in improvement of intestinal growth and function. However, application of this peptide in SBS patient as a therapeutic agent is almost impossible due to its too short half-life (~ 2 minutes). Teduglutide (Gattex^®^, Revestive^®^) is an analogue of GLP-2, which has been produced by substitution of alanine residue at position 2 with glycine. This replacement increased GLP2 half-life to more than 2 hours.^9^ Teduglutide is the first drug approved by FDA for long-term treatment of SBS and has shown effectiveness in different clinical trials.^3,10-15^ Teduglutide binds to and activates the glucagon-like peptide-2 receptor (GLP2R) for releasing intestinal mediators to increase intestinal and portal blood flow, inhibit gastric acid secretion and decrease intestinal motility.^16^

 Clinically used teduglutide is produced by both chemical synthesis (patent number: CN104418949A) and recombinant DNA technology (US Patent Number:9987334*PED), and the current study aimed to produce recombinant teduglutide in bacterial expression system. For this, teduglutide was expressed as glutathione S- transferase (GST) tagged protein and purified using affinity and size exclusion chromatographies. The secondary structure content and biological activity of teduglutide were determined by circular dichroism (CD) spectropolarimetry and cell proliferation assay. We believe this method of production can provide advantages over the costly, time-consuming, and highly polluting industrial-scale peptide synthesis, especially for long peptides such as teduglutide.

## Materials and Methods

###  Reagents

 Isopropyl β-D-1-thiogalactopyranoside (IPTG), MTT, tryptone, yeast extract and NaCl were obtained from Sigma-Aldrich (USA). Pfu DNA polymerase was obtained from BIORON GmbH, Germany. DNA ladders, BamHI, EcoRI and T4 DNA ligase were received from Fermentas (Russia). Factor Xa was purchased from New England Biolabs (USA). Primers were synthesized by Macrogen (South Korea). Gel purification kit and plasmid mini extraction kit were obtained from Qiagen (Hilden, Germany). The teduglutide coding DNA was synthesized by Geneary Biotech Co, Ltd. Glutathione Sepharose 4B was prepared from GE Healthcare Life Sciences (Sweden). Human Caco-2 epithelial cells were purchased from Pasteur Institute of Iran. BCA protein assay kit was received from Fermentas (Russia).

###  Cloning of teduglutide coding gene into pGEX-2T vector 

 The coding sequence for teduglutide along with its receptor, GLP2R, was received in pGE vector for our previously published work.^17^ Using the construct, the coding gene of teduglutide was amplified using the primers shown in Table 1. These primers were designed to include the coding DNA sequence for factor Xa recognition sequence preceding teduglutide sequence. Amplification of teduglutide coding gene and cloning it into pGEX-2T vector was performed as described previously.^18^ Briefly, teduglutide coding gene was amplified and digested using *Bam*HI and *Eco*RI restriction enzymes and then inserted into pGEX-2T vector using T4 ligase enzymatic activity. The constructed vector was amplified in *E. coli* DH5α and evaluated by PCR reactions to confirm the presence of teduglutide coding DNA as described previously.^18^ Final confirmation was carried out by DNA sequencing.

**Table 1:** Primer sequences for amplification of teduglutide coding sequence

**Sequences**	**Primers**
Forward	AATGGATCCATCGAGGGAAGGCATGGGGATGGTT
Reverse	GTCAGGAATTCTCAATCGGTAATTTTGGTCTGAATCAG

Factor Xa coding gene is bold and underlined.

###  Teduglutide expression and purification

 The process for expression and purification of teduglutide was performed as described elsewhere.^18,19^ Briefly, pGEX-2T vector harbouring teduglutide coding gene was transformed into *E. coli *BL21 (DE3) and cultured in a 200 mL LB-ampicillin (100 µg/mL) medium. At OD of 0.6, IPTG at the final concentration of 0.4 mM was used for overexpression of teduglutide attached to the C-terminus of glutathione S-transferase (GST) at 20°C while shaking at 180 rpm. The expression of teduglutide attached to GST protein was monitored at different time intervals. Overnight culture (16 hours) was harvested and resuspended in lysis buffer. The bacterial suspension was disrupted by sonication and freeze-thaw cycles and the supernatant was subjected to the Glutathione Sepharose affinity column. After 30 minutes incubation, the column was extensively washed and teduglutide was cleaved off the column by addition of 10 µg factor Xa enzyme prepared in factor Xa buffer (Tris-HCl, pH 8.0 with 100 mM NaCl and 2 mM CaCl_2_). The affinity purified teduglutide was applied to Sephacryl^®^ S-100 HR SEC column (GE Healthcare, USA) and eluted using phosphate buffer (10 mM, pH 7.4) as the mobile phase with flow rate of 0.5 mL/min. The elution fractions corresponding to teduglutide were pooled and concentrated. AKTA FPLC system (GE Healthcare, USA) was used for carrying out all chromatographies.

 The protein expression and purification in each step was monitored using 16% Tricine–SDS-PAGE under reducing conditions. BCA protein assay kit (Fermentas, USA) was used to quantify the protein concentration.

###  Circular dichroism studies

 CD experiment was conducted as explained previously.^17^ Teduglutide was prepared in 10 mM phosphate buffer (pH 7.4) at the concentration of 0.04 mg/mL and analyzed on a CD215 (Aviv, USA) spectropolarimeter. Spectra were recorded at 25°C over the wavelength range 190–260 nm using a quartz cuvette with an optical path length of 1 mm. The backgrounds were subtracted and the obtained data were converted to mean residue ellipticity (MRE) based on the following equation:

*MRE = θ*_obs_ *×*[(0.1 * *MRW*)/(*l* * c)]

*θ*_obs_ is the observed ellipticity in degrees at the defined wavelength, MRW is the mean residue weight, c is the protein concentration (mg/mL) and 0.l is the optical path (cm). Based on the obtained spectra, the secondary structure contents were analyzed using CONTIN^20,21^ and K2D3^22^ algorithms.

###  Mass spectroscopy

 To 300 µL teduglutide solution (100 µg.mL^-1^) in PBS (pH 7.4) was added 700 µL pure formic acid to obtain a solution of teduglutide containing 70% formic acid. To the reaction mixture was added cyanogen bromide (CNBr) at 100-fold molar excess with respect to a methionyl residue of teduglutide and the mixture was incubated at the room temperature for 20 hours in dark with shaking. Electrospray ionization mass spectroscopy (ESI MS) equipped with ZQ detector, a quadrupole mass analyzer (Waters, Micromass ZQ), was used for mass analysis. The spectra were recorded at the range of 300 to 2000 m/z.

###  Teduglutide proliferative effect

 Human Caco-2 epithelial cells were cultured in DMEM supplemented with 10% FBS. The cells were harvested and 10,000 cells/well were seeded in a 96-well plate and incubated for 24 hours at 37°C under a humidified atmosphere and 5% CO2. Then, cells were treated with various concentrations of teduglutide ranging from 0.0625 to 3.636 µM in serum-free medium for 72 hours. After that, 20 µL of MTT (5 mg/mL) was added to each well and the plate was incubated for additional 4 hours at 37°C under a humidified atmosphere and 5% CO2. Supernatant was discarded and to each well 100 µL of DMSO was added. Finally, the plate was incubated for 40 minutes at room temperature with agitation and the absorbances were measured at 570 nm using Epoch^TM^ Microplate Spectrophotometer (Bio Tek, USA).

## Results and Discussion

 Teduglutide (Gattex, Revestive) is an analogue of GLP-2, with a single substitution (Ala2Gly) in GLP2 to increase its half-life.^23,24^ Teduglutide is the only drug approved for long-term treatment of SBS with confirmed beneficial effects in different clinical trials.^3,10-15^ Glepaglutide (NCT03905707) and apraglutide are other analogues of GLP-2 which are in phase III clinical trials for treatment of SBS patients. Apraglutide has longer half life compared to teduglutide due to amino acid substitutions (Ala^2^ > Gly, Met^10^ > aminocaproic acid, Asn^11^ > D-Phe, Asn^16^ > Leu) in GLP-2.^25^ Teduglutide has been produced by both chemical synthesis (patent number: CN104418949A) and recombinant DNA technology (US Patent Number:9987334*PED), however, the methods for preparation are patented. Chemical synthesis is a conventional approach for peptide synthesis, however, this method suffers from serious drawbacks such as the use of strong solvents and hazardous coupling agents as well as difficulty in recycling used resins in synthesis.^26^ To bypass these difficulties alternative peptide production approaches such as recombinant DNA technology was considered which is a more convenient, cost-effective and environmentally friendly peptide production methodology.^27,28^ Based on this knowledge, the current study aimed to develop a methodology for production of teduglutide based on recombinant DNA technology. For this, teduglutide coding gene was cloned into pGEX-2T vector and overexpressed attached to GST tag protein. Therapeutic proteins require a specific N-terminus for biological activity and the cleavage of fusion proteins almost results in the generation of non-native N-terminal amino acids.^29^ Factor Xa is a proteolytic enzyme which recognizes Ile-Glu/Asp-Gly-Arg sequence and cleaves after Arg. Therefore, insertion of this sequence at the N-terminus of teduglutide and C-terminus of fusion tag (GST- Ile-Glu/Asp-Gly-Arg- teduglutide) enabled factor Xa mediated cleavage of teduglutide from the fusion partner without any extraneous residues which is suitable for the therapeutic purposes.

###  Cloning of teduglutide into pGEX-2T vector

**Figure 1:**
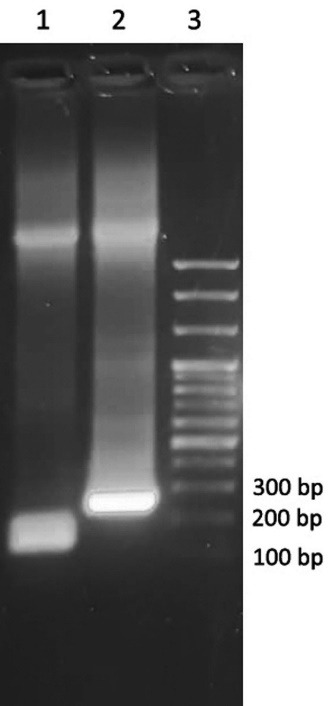
Teduglutide coding DNA amplification and confirmation of cloning by PCR reaction analyzed by gel electrophoresis. Lane 1 shows the result of amplification of teduglutide coding DNA, lane 2 is related to the PCR products on the pGEX-2T vector consisting teduglutide coding gene using pGEX universal primers. 100 bp DNA ladder used for determining the length of amplified sequence (lane 3). All the samples were run on 2% agarose gel

**Figure 2:**
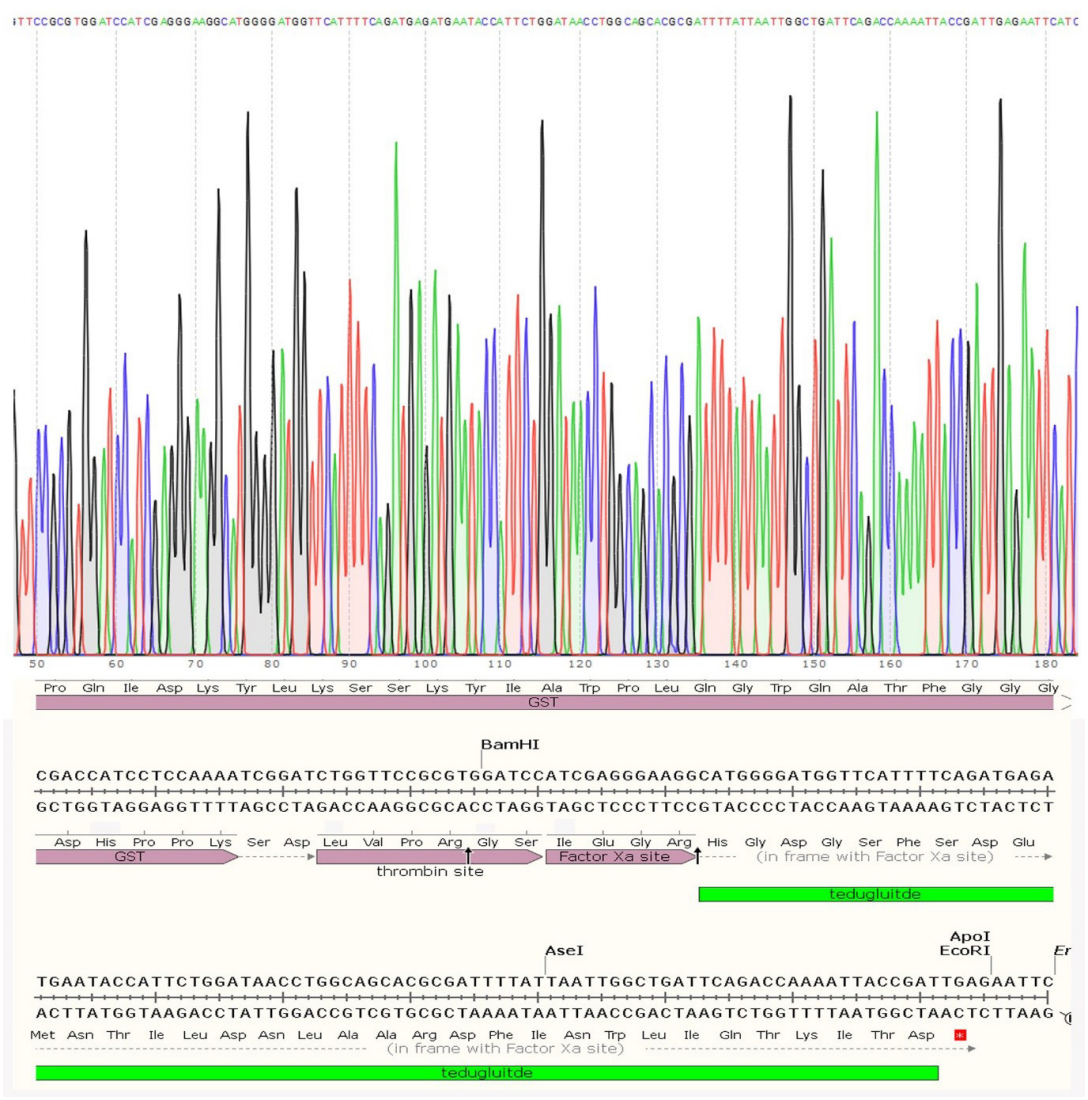
Sequencing result and the map of pGEX-2T multiple cloning site harboring teduglutide gene. As seen, the coding gene of teduglutide was preceded by factor X cleavage site coding gene

 Teduglutide coding DNA was received in pGE vector and then was amplified using the primers presented in Table 1. A DNA band with a size of 130 bp in Figure 1 (lane 1) stands for the amplified teduglutide. The PCR product was digested and cloned into the pGEX-2T vector using T4 DNA ligase enzymatic activity. A PCR reaction using the universal primers of pGEX vectors (forward: 5’GGGCTGGCAAGCCACGTTTGGTG3’ and reverse: 5’CCGGGAGCTGCATGTGTCAGAGG3’) was carried out on the constructed vector to validate the insertion of teduglutide coding sequence (Figure 1). Further confirmation was carried out by sequencing indicating that teduglutide coding gene has been successfully cloned into MCS of pGEX-2T vector (Figure 2).

###  Expression and purification of teduglutide 

**Figure 3:**
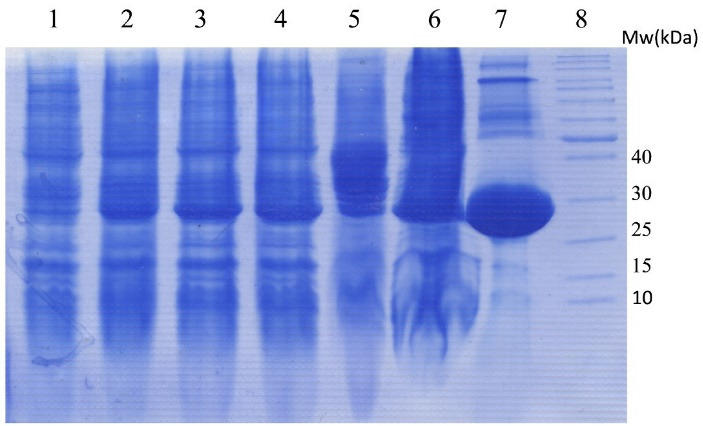
The SDS-PAGE gel electrophoresis of GST-teduglutide fusion protein produced in *BL21(DE3)*. The lanes are samples prepared from soluble fraction of bacterial lysates: (1) before induction, (2) 1 hour, (3) 2 hours, (4) 3 hours and (5) overnight incubation after induction by 0.4 mM IPTG, (6) bacterial pellet, (7) GST-teduglutide fusion protein purified using glutathione sepharose bead (8) Protein ladder. The protein band of 29.6 kDa is related to GST-teduglutide fusion protein.

**Figure 4:**
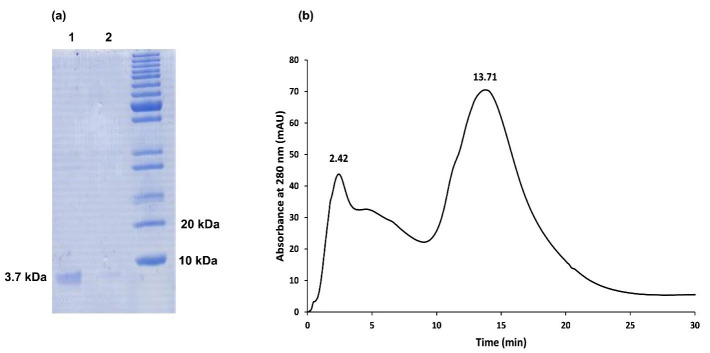
The process for GLP2R-teduglutide purification. In panel (a), lanes 1 and 2 stand for the Tricine–SDS-PAGE analysis of teduglutide (3.75 kDa) resulted from the affinity purification and size exclusion chromatography, respectively. The spectrum for the size exclusion chromatography analysis is presented in panel (b) where the peak observed at 13.71 min was related to teduglutide

 The expression of teduglutide attached to GST protein was carried out in *E. coli *BL21 (DE3). The expression was induced by adding IPTG followed by incubation overnight in a shaker-incubator set to 20°C. Samples were taken before and after induction and analyzed on SDS-PAGE. As shown in Figure 3 the GST-teduglutide fusion protein was overexpressed as a function of the inducer and reached to the maximum at overnight incubation. The soluble fraction obtained from bacterial disruption was subjected to Glutathione Sepharose 4B gel affinity column to bound and separate GST-teduglutide fusion protein from the rest of bacterial proteins. Using factor Xa proteolytic activity teduglutide was cleaved off the column bound fusion partner. The collected sample resulted in a 3.7 kDa protein band on Tricine-SDS-PAGE gel which was attributed to teduglutide (Figure 4a). To get rid of factor Xa, the affinity purified sample was subjected to size exclusion chromatography on a Sephacryl^®^ S-100 HR column (Figure 4b), where a peak observed at retention time of 13.71 min was correlated with teduglutide. The total protein of soluble fraction and purified teduglutide was measured using BCA protein assay kit. The results are available in Table 2 where from 200 mL bacterial culture, 14.67 mg total protein and 0.45 mg pure teduglutide was obtained after two steps of purification. These values are comparable with those reported for the peptides with the sizes close to teduglutide.^30-33^

**Table 2:** Purification process of teduglutide from 200 mL culture of *E. coli BL21 (DE3) *transformed with pGEX-2T vector containing teduglutide gene

**Culture volume**	**Bacterial mass**	**Total protein mass in soluble fraction**	**Total protein mass after affinity purification**	**Total protein mass (teduglutide) after Size exclusion chromatography**
200 mL	1.50 g	14.67 mg	1.00 mg	0.45 mg

Protein concentration was measured using BCA protein assay kit.

###  Investigation of teduglutide secondary structure content

**Figure 5:**
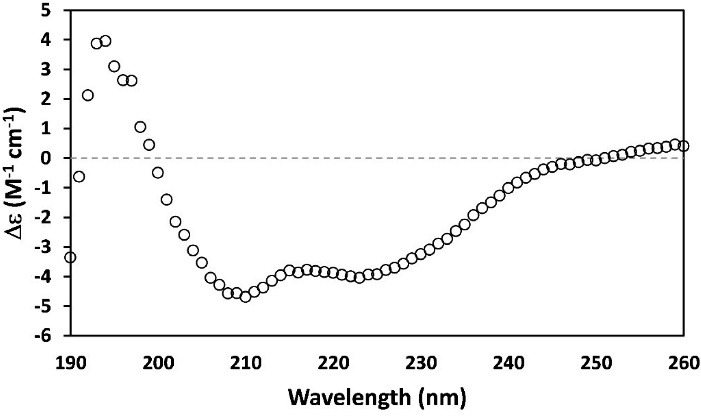
CD spectrum of teduglutide (0.04 mg/mL) recorded in 10 mM phosphate buffer (pH 7.4) at 25°C. The percentages of alpha helix secondary structure estimated using CONTIN and K2D3 programs were 65% and 51.10%, respectively

 Proper folding of teduglutide obtained from SEC was assessed by determining its secondary structure content using CD spectroscopy method. The resultant CD spectrum for teduglutide was typical for a protein with high alpha helix content^34,35^ with a positive peak at 193 nm and two negative peaks at 208 and 222 nm (Figure 5). The analyses of the CD data using CONTIN^20,21^ and K2D3^22^ methods revealed 65% and 51.10% alpha helix content in purified teduglutide, respectively. These values are in agreement with the result of Jpred^36^ and PSIPRED^37^ secondary structure prediction tools (66.67% alpha helix content) and NMR solution structure of GLP-2 in 2,2,2 trifluoroethanol (PDB ID:2L63) (72.7%),^38^ indicating that the produced teduglutide was folded properly.

###  Mass spectroscopy analysis of the produced teduglutide

**Figure 6:**
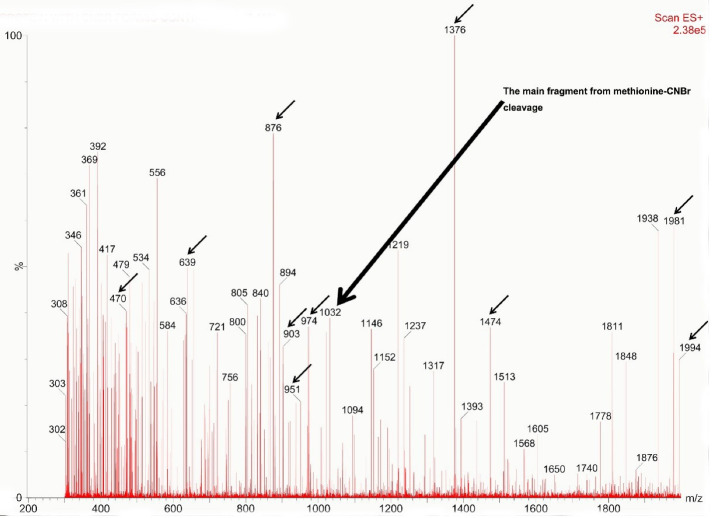
The mass spectrum regarding the teduglutide treated with CNBr. Some theoretically calculated masses were highlighted in the figure and their corresponding fragments are available in Supplementary data

**Figure 7:**
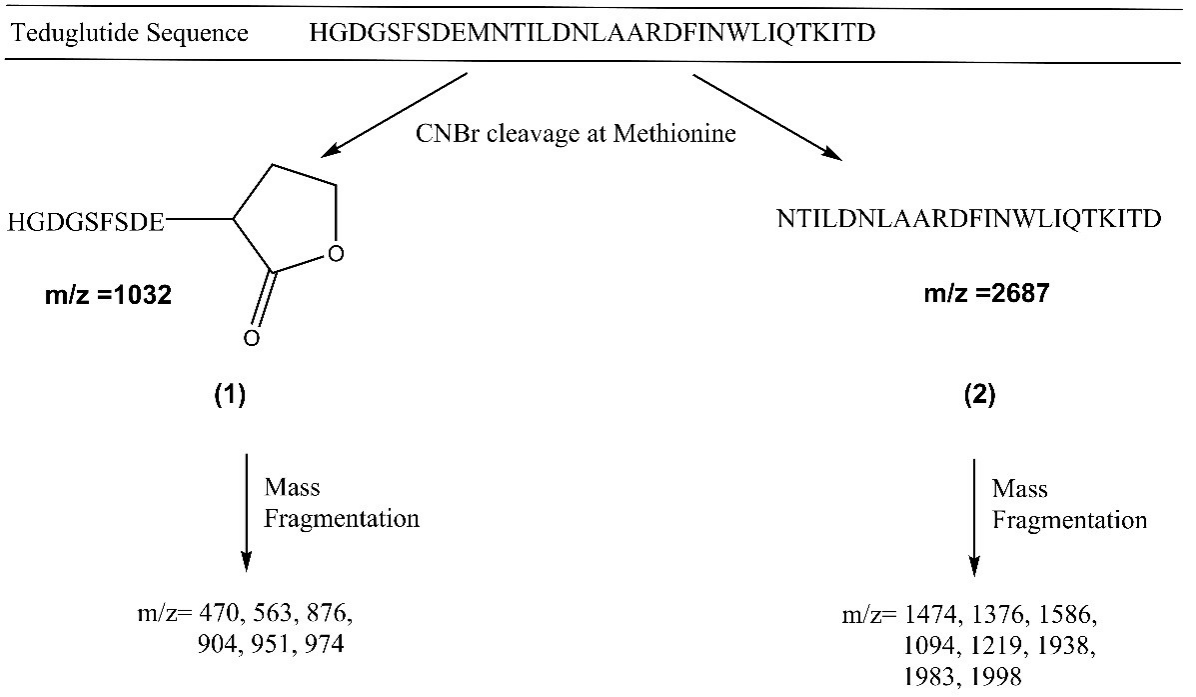
The fragments of teduglutide resulted from CNBr cleavage indicated as fragments 1 and 2. The m/z of fragments resulted from mass fragmentation of fragments 1 and 2 have also been provided

 Mass spectroscopy is a valuable technique for analysis of biomolecules such as proteins and peptides. Here, it was used to characterize the produced teduglutide. For this, a solution of teduglutide in formic acid was prepared and CNBr mediated cleavage was carried out prior to mass spectroscopy analysis. Figure 6 illustrates the obtained spectrum for CNBr treated teduglutide carried out using ESI technology at the range of 300 to 2000 m/z. The cleavage of methionine-containing teduglutide with CNBr generated a C-terminal homoserine lactone residue (fragment 1), which was detected in mixture in mass spectroscopy with m/z of 1032 (Figure 7). Also, mass fragmentations of this lactone-containing fragment was appeared at m/z of 470 (469 + 1H), 563 and 876 (1032 – CO_2_H -C_4_H_6_NO_2 _+ 2H) as well as 951, 974. Due to upper limit of measurable mass of 2000 m/z by our mass spectrometer, one of the expected teduglutide fragments due to CNBr cleavage, fragment 2 (i.e., NH_2_-Asn-Thr-Ile-Leu-Asp-Asn-Leu-Ala-Ala-Arg-Asp-Phe-Ile-Asn-Trp-Leu-Ile-Gln-Thr-Lys-Ile-Thr-Asp) (m/z 2687), was not detectable. However, its fragments with m/z less than 2000 were detected. The most important fragment which indicates the presence of fragment 2 in CNBr treated teduglutide sample was a peak at m/z of 1376. The possible other fragments were also analyzed and indicated as fragments 2a to 2f in Supplementary File 1. All these observations proved successful production of teduglutide using the procedure presented in the current study.

###  Assessment of teduglutide proliferative activity

**Figure 8:**
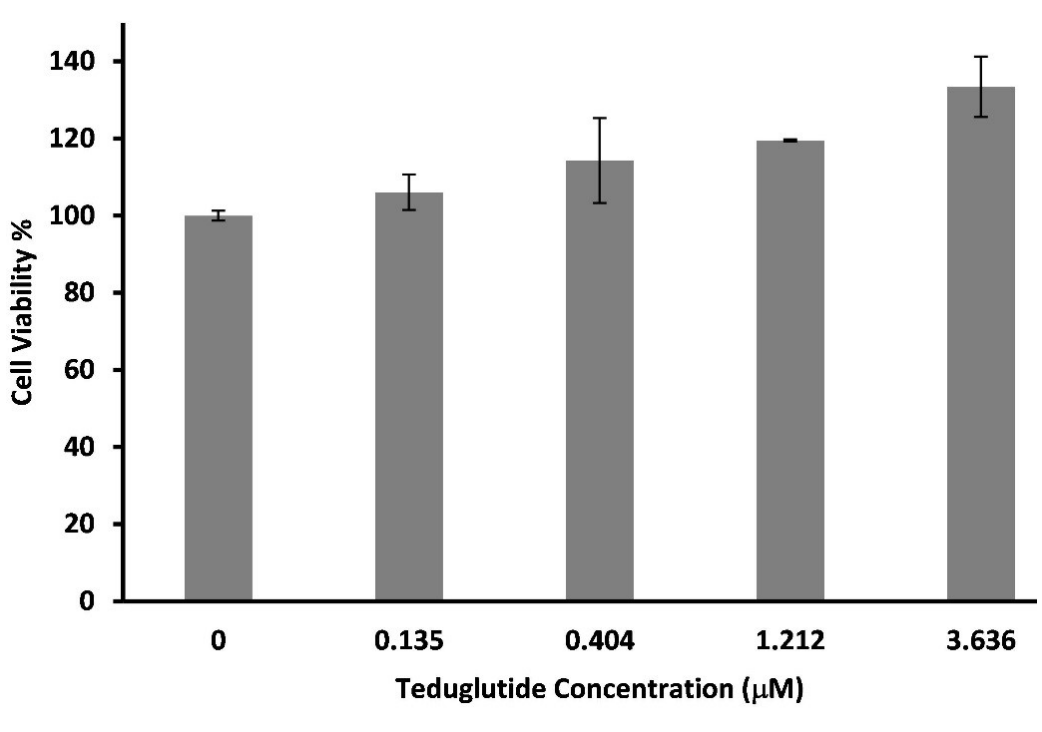
Effect of teduglutide on human Caco-2 intestinal epithelial cell proliferation. Cells were treated with various concentrations of teduglutide ranging from 0.0625 to 3.636 µM in serum-free medium for 72 h. At the concentrations of 3.64 and 1.21 µM, teduglutide is capable to induce cell proliferation by 33 ± 7.82 and 19 ± 0.30 %, respectively, compared to untreated cells

 In order to determine the activity of produced teduglutide, MTT assay was employed where different concentrations of teduglutide ranging from 0 to 3.64 µM was added on human Caco-2 epithelial cells and the percentages of cell proliferation were assessed (Figure 8). The results showed that, at the concentrations of 3.64 and 1.21 µM, teduglutide is capable to induce cell proliferation by 33 ± 7.82 and 19 ± 0.30 %, respectively, compared to untreated cells (zero concentration of teduglutide) indicating that the obtained teduglutide is biologically active. These observations were consistent with the result of a study in which teduglutide caused 10 % more Caco-2 cell proliferation at the concentration of 0.5 µM compared to untreated cells.^39^

 Harnessing the advantages of recombinant DNA technology led to first ever manufactured recombinant protein, insulin, by Genentech in 1982 and after that more and more therapeutic proteins and peptides such as calcitonin, ecallantide and teduglutide were produced recombinantly.^27,28^ The results obtained in the current study indicate successful production of teduglutide which is correctly folded and is active in a biological *in vitro* experiment. It is believed that the procedure presented in the current study can be scaled up for large scale production of teduglutide and also can be generalized to other therapeutic peptides.

## Conclusion

 Teduglutide is the first and only therapeutic indicated for long-term treatment of SBS. In the current study, teduglutide was expressed as a GST-tagged fusion protein in bacterial expression system. Upon activity of factor Xa, teduglutide was cleaved off from the fusion partner. The affinity purified peptide was further purified using size exclusion chromatography and its correct folding and activity were verified using CD and cell proliferation assays. We believe that the experimental-scale method presented in the current study can be useful for pilot-scale and also industrial-scale production of teduglutide and other peptides and proteins.

## Acknowledgments

 This work was supported by national institute for medical research development (NIMAD) (Grant number 977415). The authors would like to thank Biotechnology Research Center of Tabriz University of Medical Sciences for providing facility support.

## Competing Interests

 The authors declare no conflict of interest.

## Ethical Approval

 Not applicable.

## Supplementary Files

Supplementary file 1. Main fragments from reaction of cyanogen bromide with methionine residue
