# Supplementary material for: Expression, Purification and Characterization of Functional Teduglutide Using GST Fusion System in Prokaryotic Cells
Source: Adv Pharm Bull. 2022 Dec 6;13(3):592–600. doi: 10.34172/apb.2023.064 (PMC10460804; doi:10.34172/apb.2023.064)
Supplement: Supplementary file 1 — Main fragments from reaction of cyanogen bromide with methionine residue [file apb-13-592-s001.pdf]

Adv Pharm Bull, 2023, 13(3), 592-600
doi: 10.34172/apb.2023.064
https://apb.tbzmed.ac.ir

Main fragments from reaction of cyanogen bromide with methionine residue

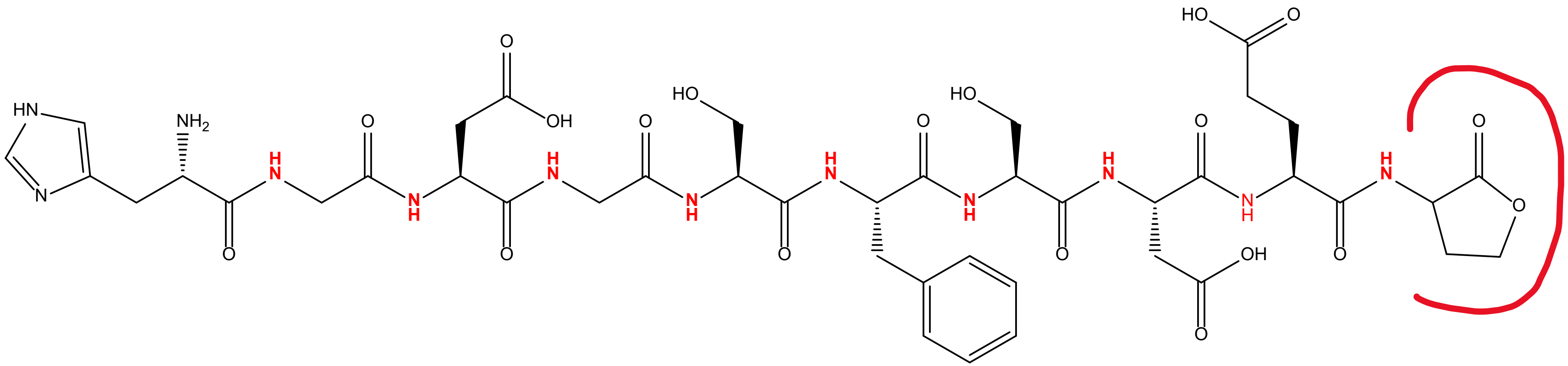

Chemical Formula: $C_{42}H_{56}N_{12}O_{19}$
Exact Mass: 1032.38
Molecular Weight: 1032.98

Compound (1)

Asn-Thr-Ile-Leu-Asp-Asn-Leu-Ala-Ala-Arg-Asp-Phe-Ile-Asn-Trp-Leu-Ile-Gln-Thr-Lys-Ile-Thr-Asp

Chemical Formula: $C_{121}H_{194}N_{32}O_{37}$
Exact Mass: 2687.43
Molecular Weight: 2689.07

Compound (2)

- **Mass Fragmentation of Compound (1)**

1- m/z= 469 AND 563

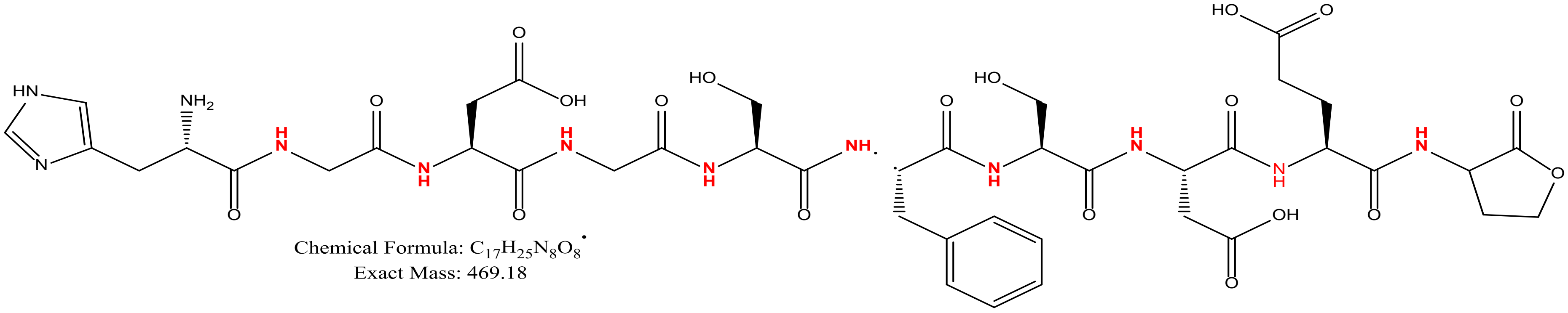

Chemical Formula: $C_{17}H_{25}N_8O_8^{\bullet}$
Exact Mass: 469.18

Chemical Formula: $C_{25}H_{31}N_4O_{11}^{\bullet}$
Exact Mass: 563.20

(1a) **(1b)**

2- m/z= 904 AND 128

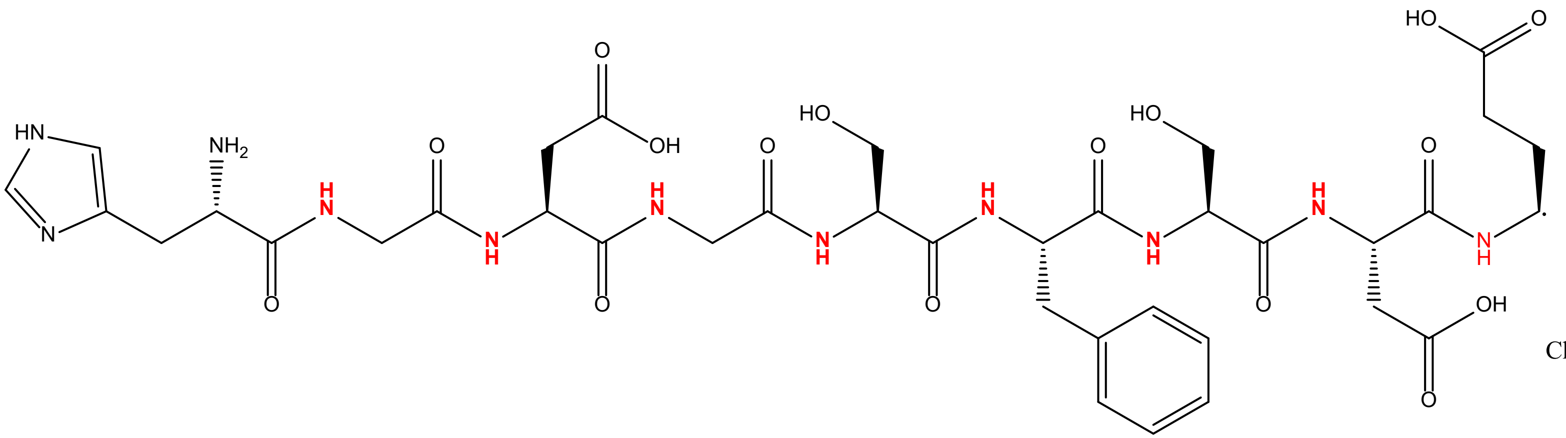

Chemical Formula: $C_{37}H_{50}N_{11}O_{16}^{\bullet}$
Exact Mass: 904.34

(1c)

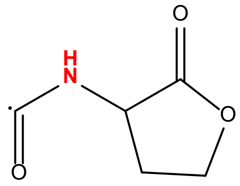

Chemical Formula: $C_{5}H_{6}NO_{3}^{\bullet}$
Exact Mass: 128.03

(1d)

3- m/z= 876 (873+ 2H) + 1H

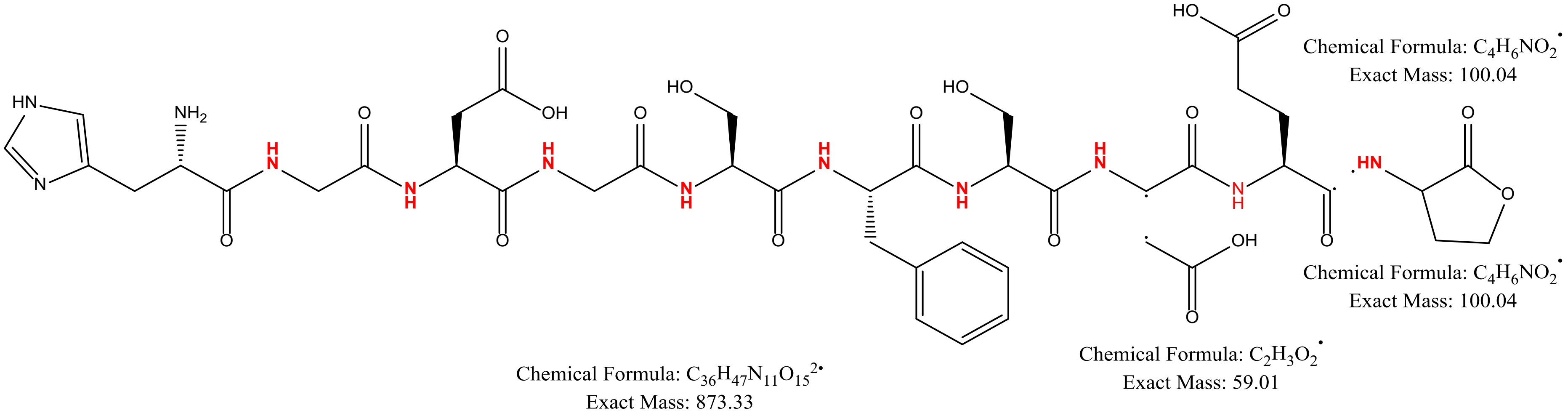

(1e) **(1f)** **(1g)**

4- m/z= 951

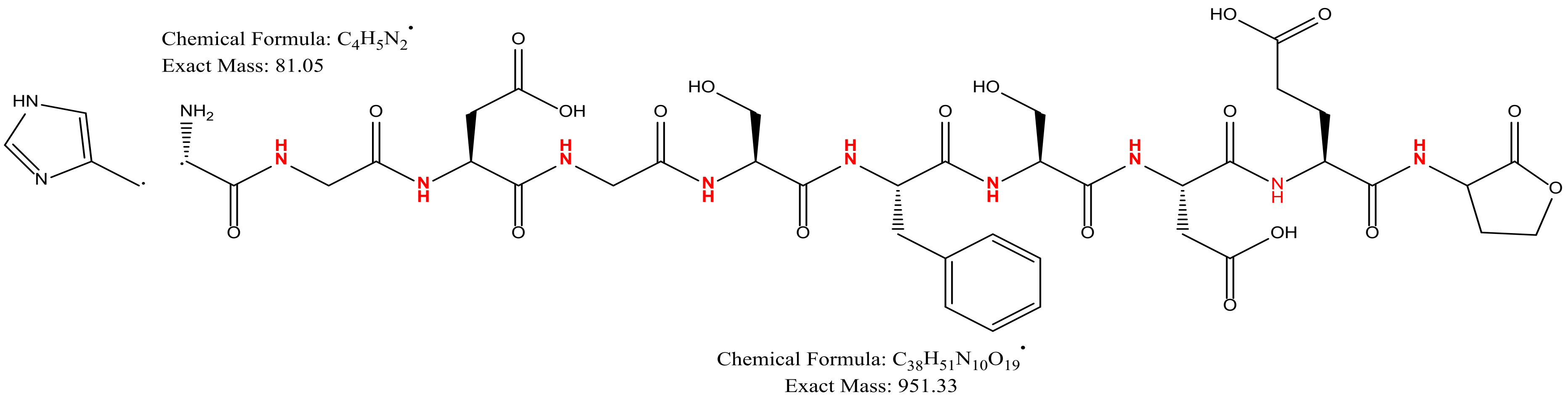
Chemical Formula: $C_4H_5N_2^{\bullet}$
Exact Mass: 81.05
HN
N
NH_2
H
N
O
OH
HO
Chemical Formula: $C_{38}H_{51}N_{10}O_{19}^{\bullet}$
Exact Mass: 951.33

(1h)

5- m/z= 974

Chemical Formula: $C_2H_4O_2$
Exact Mass: 60.02
Molecular Weight: 60.05

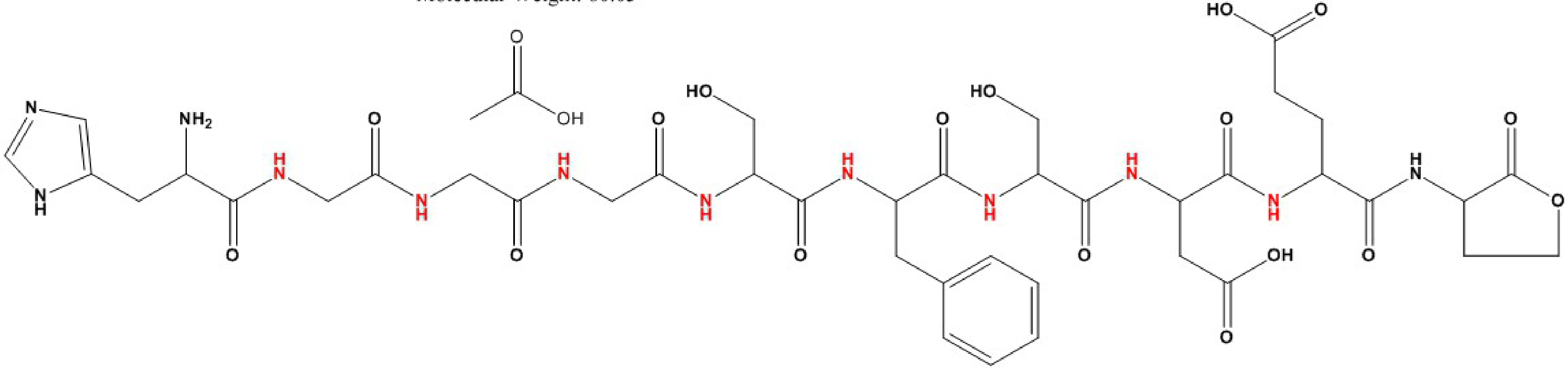

Chemical Formula: $C_{40}H_{54}N_{12}O_{17}$
Exact Mass: 974.37
Molecular Weight: 974.94

(1i)

- **Mass Fragmentation of Compound (2)**

1- m/z= 1376 (1374+2H)

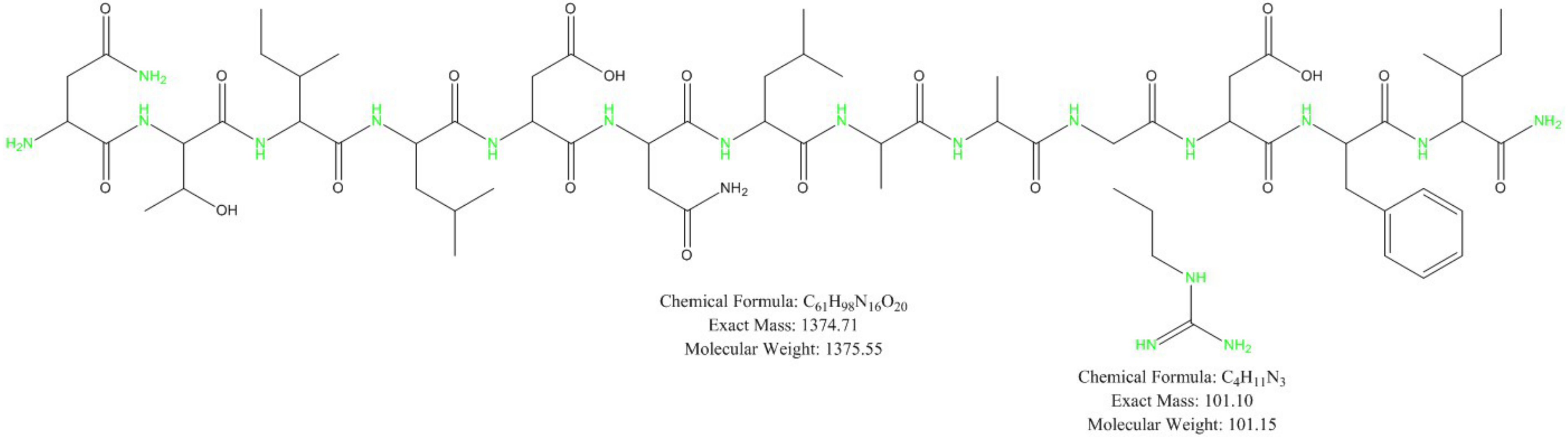

(2a)

2- m/z= 1474

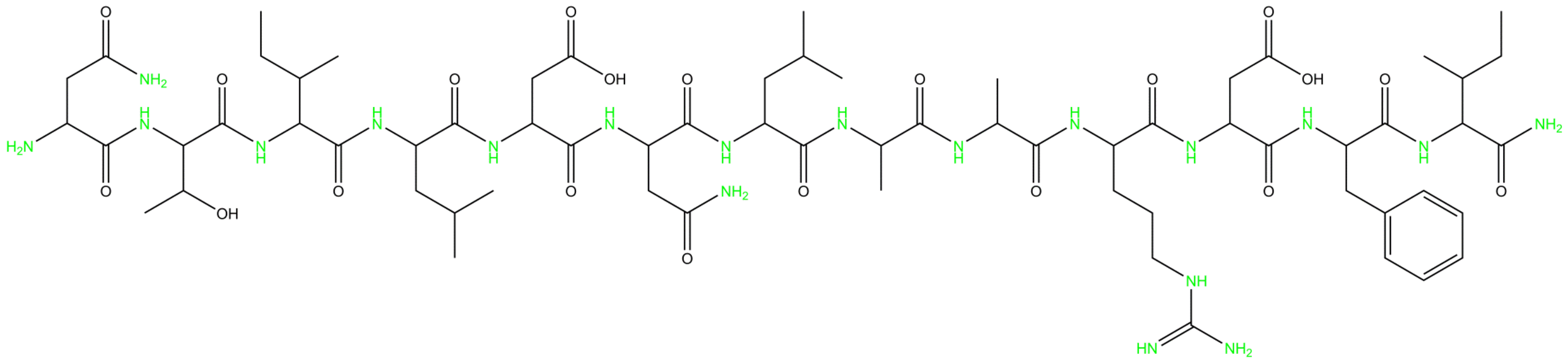

Chemical Formula: $C_{65}H_{107}N_{19}O_{20}$
Exact Mass: 1473.79
Molecular Weight: 1474.68

(2b)

3- m/z= 1219 (1216+3H)

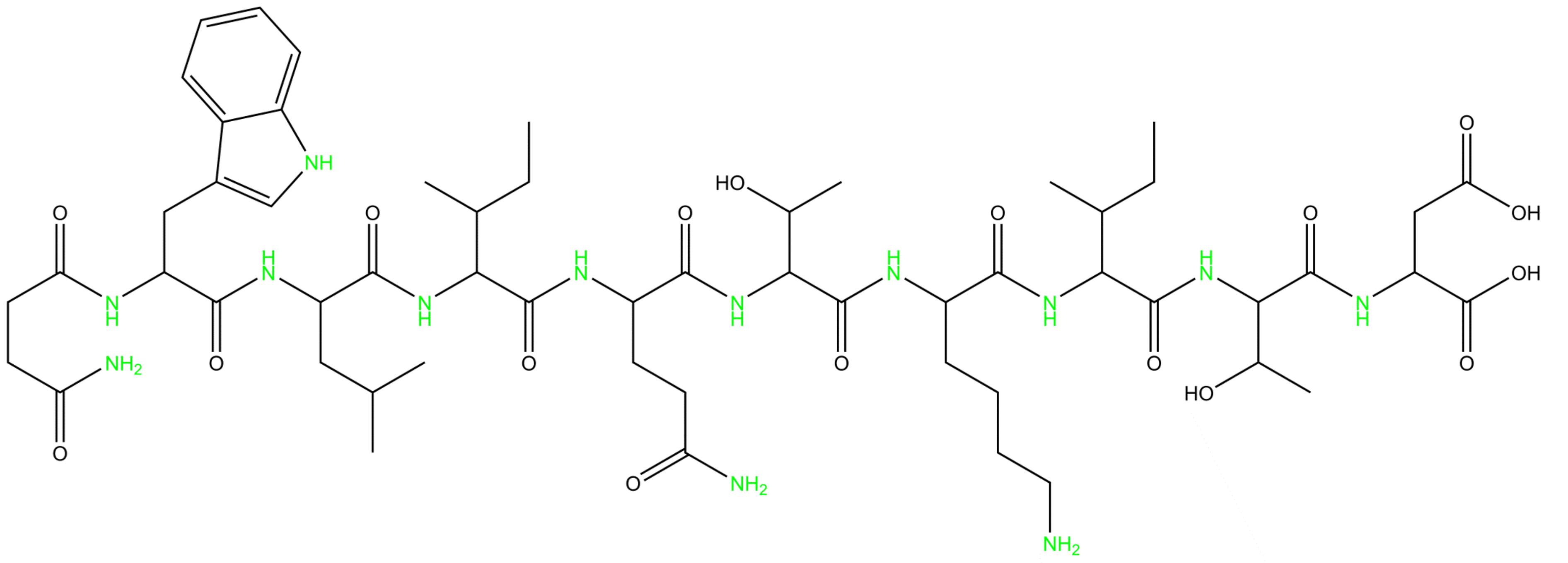

Chemical Formula: $C_{56}H_{89}N_{13}O_{17}$
Exact Mass: 1215.65
Molecular Weight: 1216.40

(2c)

4- m/z= 1998

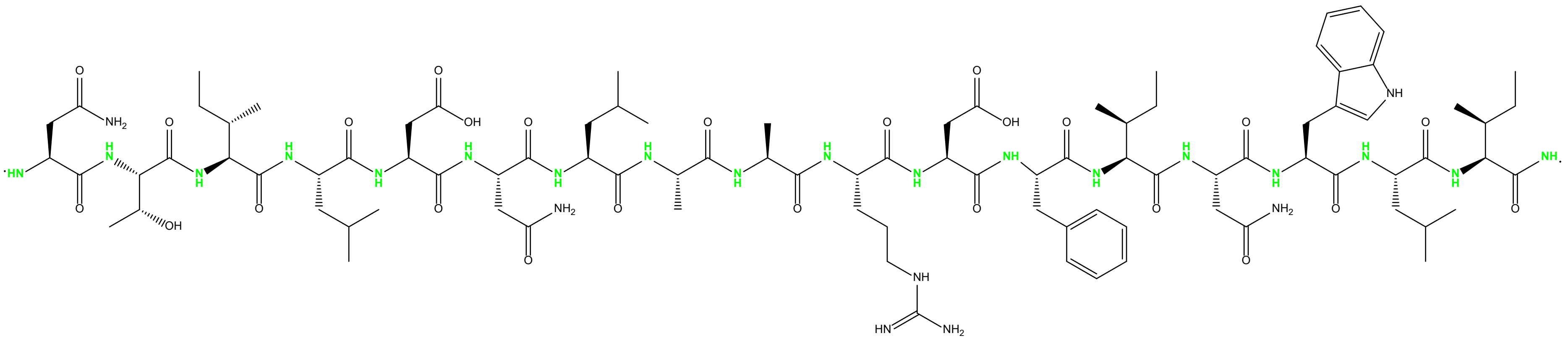

Chemical Formula: $C_{92}H_{143}N_{25}O_{25}^{2\bullet}$
Exact Mass: 1998.07

(2d)

5- m/z= 1981 (1993-2H)

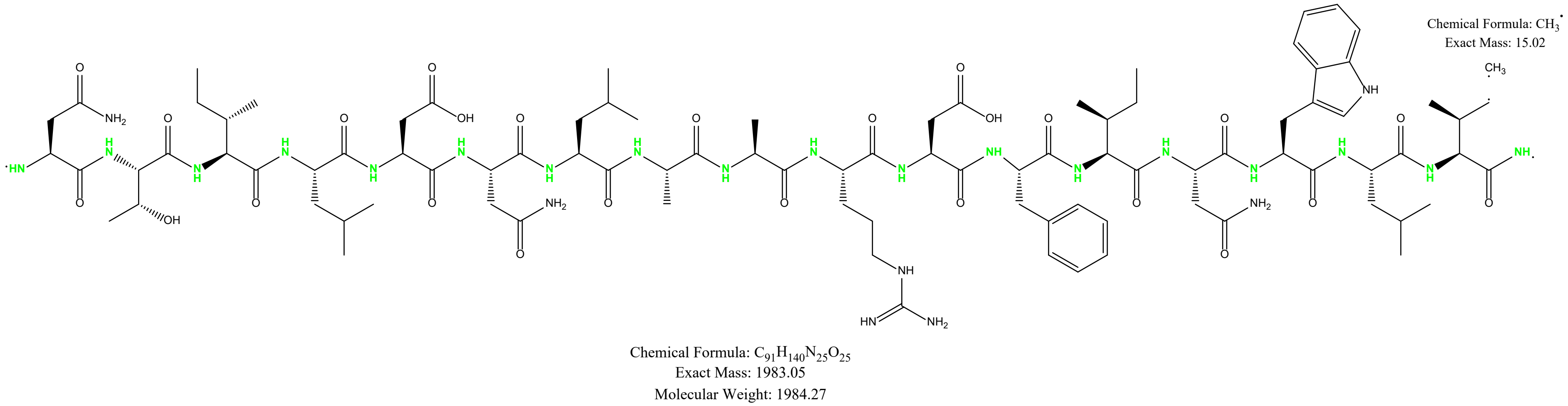

(2e)

6- m/z= 1938 (1940-2H)

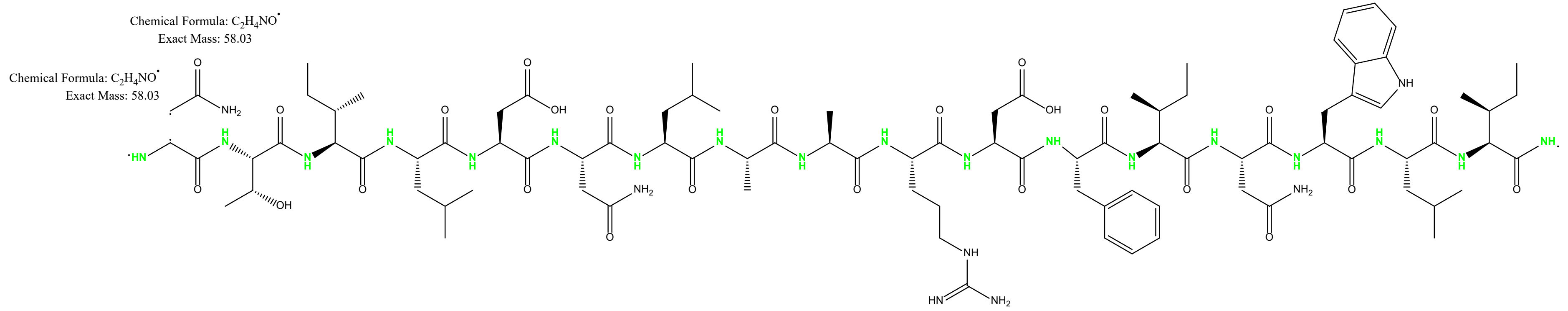

Chemical Formula: $C_{90}H_{139}N_{24}O_{24}$
Exact Mass: 1940.04
Molecular Weight: 1941.25

(2f)
